# Tailoring the Composition of Ba_x_BO_3_ (B = Fe, Mn) Mixed Oxides as CO or Soot Oxidation Catalysts in Simulated GDI Engine Exhaust Conditions

**DOI:** 10.3390/molecules28083327

**Published:** 2023-04-09

**Authors:** Álvaro Díaz-Verde, Salvador Montilla-Verdú, Verónica Torregrosa-Rivero, María-José Illán-Gómez

**Affiliations:** Carbon Materials and Environment Research Group, Inorganic Chemistry Department, University of Alicante, 03690 Alicante, Spain; alvaro.diaz@ua.es (Á.D.-V.); salvador.montilla@ua.es (S.M.-V.); vero.torregrosa@ua.es (V.T.-R.)

**Keywords:** perovskite, iron, manganese, CO oxidation, GDI soot oxidation

## Abstract

Mixed oxides with perovskite-type structure (ABO_3_) are promising catalysts for atmospheric pollution control due to their interesting and tunable physicochemical properties. In this work, two series of Ba_x_MnO_3_ and Ba_x_FeO_3_ (x = 1 and 0.7) catalysts were synthesized using the sol–gel method adapted to aqueous medium. The samples were characterized by μ-XRF, XRD, FT-IR, XPS, H_2_-TPR, and O_2_-TPD. The catalytic activity for CO and GDI soot oxidation was determined by temperature-programmed reaction experiments (CO-TPR and soot-TPR, respectively). The results reveal that a decrease in the Ba content improved the catalytic performance of both catalysts, as B0.7M-E is more active than BM-E for CO oxidation, and B0.7F-E presents higher activity than BF for soot conversion in simulated GDI engine exhaust conditions. Manganese-based perovskites (BM-E and B0.7M-E) achieve better catalytic performance than iron-based perovskite (BF) for CO oxidation reaction due to the higher generation of actives sites.

## 1. Introduction

Currently, limitation of climate change is one of the most challenging topics due to its dangerous global effects on biological and human systems [1]. Greenhouse gases (GHGs), such as carbon dioxide, methane, and nitrogen oxides, are among the main species causing this problem. Since the industrial revolution, GHGs have been emitted into the atmosphere as the result of human activities, and their release has continuously increased [2]. The transport sector is the most responsible for GHG emissions, and predictions indicate that vehicles will contribute around 85% of the total GHGs emissions in 2040 if effective mitigation measures are not applied [3,4]. This scenario is pushing to the automotive industrial sector to use alternative systems to internal combustion engines, such as electric and fuel-cell-based engines [5,6,7]. However, nowadays, electric and fuel-cell-based vehicles are not as available as internal-combustion-engine-based vehicles due to their high economical costs, the limited number of available charging points, and their long charge times [8,9]. For this reason, internal combustion systems cannot be totally eliminated in the coming years, and consequently, additional measures to reduce the amount of pollutants emitted by these engines must continue to be implemented.

In addition to the GHGs, carbon monoxide (CO) and soot are considered dangerous pollutants, as they also increase morbidity rates. These chemical species, which are highlighted as hazardous emissions generated by gasoline direct injection (GDI) engines, have become more relevant in recent last years [10,11]. As GDI-engine-based vehicles offer a high fuel economy and reduced CO_2_ emissions (compared to other gasoline engines), they were the most widely purchased passenger cars in 2017 [12].

The removal of these two pollutants is carried out using catalysts (three-way catalysts (TWCs) and gasoline particulate filters (GPF) units, respectively) that are able to convert these toxic substances into more benign ones. However, to comply with current and future legislation and due to the working conditions of current internal combustion engines, the development of a new generation of active phases for TWC and GPF units is a challenging task. These new active phases, among other characteristics, should present higher activities at lower temperatures than the current active phases. Noble-metal/ceria-based catalysts are proposed as promising materials for these applications due to the high oxygen storage capacity (OSC) and good redox properties of ceria-based support. However, these solids have limited use under real conditions, mainly due to the high cost of noble metals, (the amount of which should be minimized as much as possible [13,14,15]) and their low stability under working conditions for long periods of time [10]. In this sense, noble-metal-free catalysts, such as as mixed oxides with perovskite-type structure (ABO_3_) [16,17,18,19,20], have been proposed as an interesting family of catalysts combining tunable catalytic properties and low cost.

It is well known that the redox properties of the active phase of catalysts play an essential role in oxidation reactions, such as CO and soot. In fact, perovskites stand out because of their excellent redox properties, which are mainly determined by B cations. A problem associated with using noble-metal-based catalysts for CO oxidation is the inhibition caused by CO adsorption (which hinders the dissociative adsorption of O_2_ [21]). In previous papers, manganese-based perovskite solids have been proposed as catalysts to be tested for CO oxidation [22,23,24,25,26,27]. Manganese is considered an essential component of CO oxidation catalysts, as its molecular orbitals have suitable energy for the adsorption and activation of CO and O_2_ [22,23]. Moreover, the activity of CO oxidation is strongly correlated with Mn-O bond strength and increases as Mn-O bond strength decreases [22,23,24].

The CO oxidation mechanism has been widely studied, with researchers reporting that at temperatures below 300 °C, CO oxidation follows a suprafacial mechanism. Thus, the catalyst provides adsorption sites, exhibiting the appropriate electronic properties and symmetry to activate the reactants [23,25]. In fact, for transition-metal based catalysts, an Eley–Rideal mechanism has been proposed, whereby CO reduction takes place in the adsorbed states with dissociated adsorbed oxygen species [22,26,27]. At higher temperatures (T > 300 °C), the bulk lattice oxygen of the catalyst is involved in the reaction, so a Mars-Van–Krevelen redox mechanism takes place. Under such conditions, the consumed lattice oxygen is regenerated through the adsorption, dissociation, and incorporation of oxygen from the gas phase [23,25,28]. Moreover, it was found that the CO:O_2_ ratio can promote a specific mechanism and that the kinetics of the reaction strongly depend on O_2_ and CO concentrations [29], as well as on the amount of oxygen vacancies [22].

Considering this background, in order to study the CO oxidation reaction in this paper, two atmospheres were employed: (i) 0.1% CO—1% O_2_, which was selected to avoid competition for the catalyst active sites due to the low concentration of reactants, and (ii) 1% CO—1% O_2_, which was used as a closer approximation of the CO and O_2_ concentrations in GDI exhaust.

On the other hand, it has been widely reported that soot oxidation can be catalyzed by O_2_, by NO, and by a mixture of them [27,30,31,32]. However, the composition of GDI exhaust is low in NO_x_ content, as fuel cuts (i.e., when the engine pumps air forms the intake to the exhaust [33,34]) are the only source of oxygen, most of which is used by the TWC unit. Therefore, a good catalyst for GDI soot oxidation should be able to adsorb oxygen in fuel-cut mode and to desorb it under regular operating conditions. Therefore, it is interesting to analyze soot oxidation under two different atmospheres: (i) in an inert atmosphere (He), simulating the regular operation conditions of a GDI engine, and (ii) in a slightly oxidant atmosphere (1% O_2_/He), which reproduces severe fuel-cut operation conditions, as well as the typical O_2_ concentration at the turbine-GDI engine exit, i.e., upstream of the TWC [35]. 

Perovskite-based catalysts show a good oxygen storage capacity, since O_2_ can be adsorbed and activated on the oxygen vacancies [30]. Therefore, BaMnO_3_ solids have been used for GDI soot oxidation under the two different atmospheres indicated above [36], as well as for CO oxidation in simulated GDI exhaust engine conditions [37]. Additionally, we recently realized that the use of under-stoichiometric Ba_x_MnO_3_ perovskites improves the performance of raw BaMnO_3_ for CO oxidation, showing that Ba_0.7_MnO_3_ formulation achieves the best results [38]. Based on this statement and the background described above for CO and soot oxidation, in this study, two series of BaBO_3_ perovskite-type catalysts (with Ba_x_MnO_3_ and Ba_x_FeO_3_ formulation of x = 1 and 0.7, respectively) were synthesized, characterized, and tested for CO and soot oxidation reactions as model reactions relevant for the potential use of these catalysts in the control of pollutants generated by GDI engines.

## 2. Results and Discussion

### 2.1. Chemical, Morphological, and Structural Characterization

**Figure 1:**
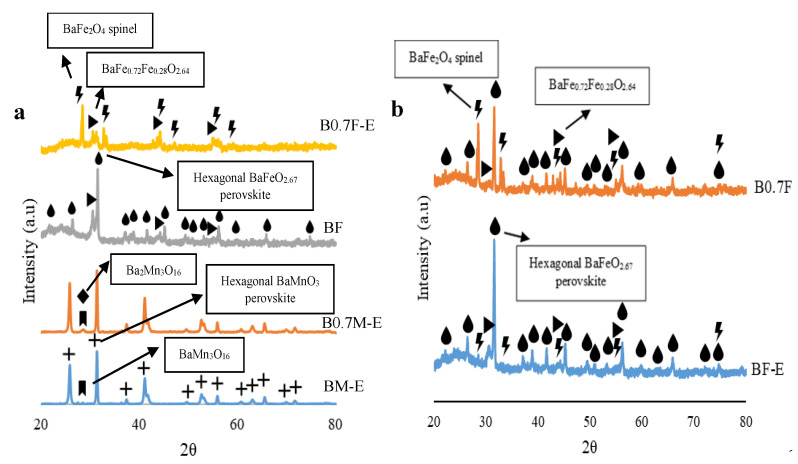
XRD patterns of (**a**) BM-E, B0.7M-E, BF, and B0.7F-E; and (**b**) BF-E and B0.7F catalysts.

X-Ray patterns for BM-E, B0.7M-E, BF, and B0.7F-E catalysts are shown in Figure 1a, and the corresponding BF-E and B0.7F samples are presented in Figure 1b. The corresponding XRD data are included in Table 1. 

For manganese-based samples, BM-E and B0.7M-E catalysts exhibit a hexagonal BaMnO_3_ perovskite structure (a = b ≠ c) as the main crystalline phase, and BaMn_3_O_16_ and Ba_2_Mn_3_O_16_ were detected as minority crystalline phases, appearing in very low amounts. The presence of these minority phases seems not to be related to the modification of the barium content, as a clear trend with barium content is not observed. Thus, the presence of these minority phases may be related to [39] (i) the formation of small precipitates during the gelation step, (ii) the use of a low calcination temperature, or (iii) a very short calcination step. Note that the decrease in barium percentage does not generate significant structural modifications, since the cell parameters are not appreciably different (Table 1). 

For iron-based samples, the BF diffractogram shows peaks corresponding to a hexagonal BaFeO_2.67_ perovskite structure (a = b ≠ c), while a peak associated with a BaFe_2_O_4_ orthorhombic spinel phase (a ≠ b ≠ c) was observed for the B0.7F-E catalyst. M. Sun et al. [40] suggested that the BaFe_2_O_4_ structure is formed when FeO_6_ octahedrons of the perovskite lattice lose oxygen to form the FeO_4_ tetrahedra present in the spinel lattice. Additionally, BaFe_0.72_Fe_0.28_O_2.64_ mixed oxide (in which Fe(III) and Fe(IV) ions should coexist) was detected as a minority phase in both samples. In Figure 1b, it is observed that BF-E and B0.7F samples present a mixture of the three crystalline phases previously described for iron-based samples, that is, hexagonal BaFeO_2.67_ perovskite, BaFe_2_O_4_ orthorhombic spinel, and BaFe_0.72_Fe_0.28_O_2.64_; however, a predominant phase is not detected. Therefore, in order to simplify, only the samples presenting a clear main crystalline phase, that is, BM-E, B0.7M-E, BF, and B0.7F-E, were selected to carry out the activity studies. 

For the selected catalysts, the average crystal size of the main crystalline phases was calculated by employing the Williamson–Hall method [41]; according to the results presented in Table 1, crystal growth is favored for stoichiometric perovskites, that is, for BM-E and BF.

In Table 2, the BET surface area (determined by N_2_ adsorption) and the chemical bulk composition (both experimental and nominal values calculated using BaMnO_3_, Ba_0.7_MnO_3_, BaFeO_2.67_, and BaFe_2_O_4_ formulae) are included for selected samples. Note that (i) all catalysts present a low surface area, as perovskite-type oxides are solids with negligible porosity [16,42,43,44,45], and (ii) the nominal and experimental bulk chemical compositions are similar, so the samples achieve the required weight percentages of Ba, Mn, Fe, or O.

**Figure 2:**
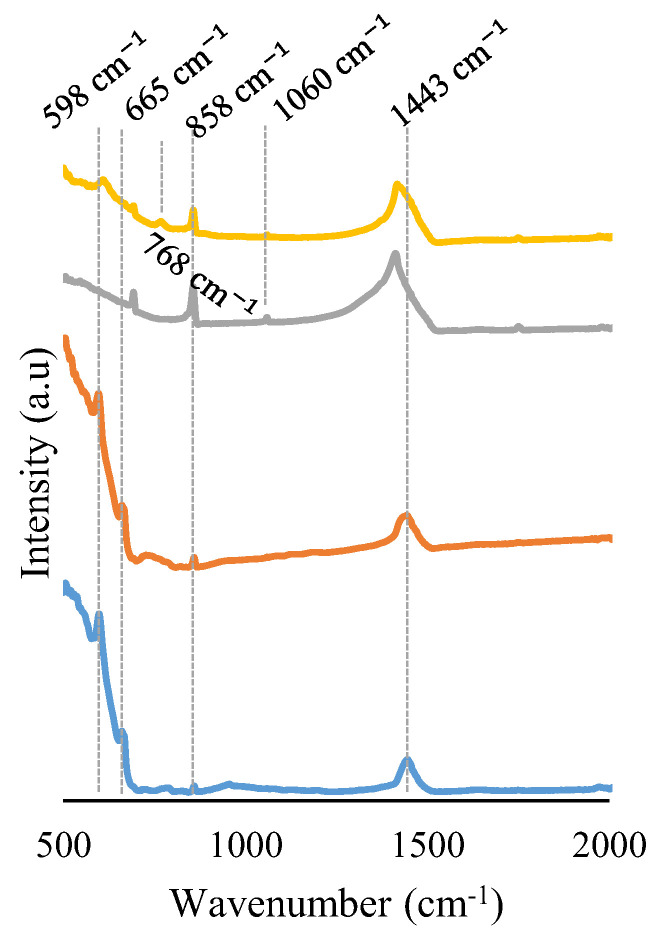
FT-IR spectra of BM-E, B0.7M-E, BF, and B0.7F-E catalysts.

FT-IR spectra of catalysts are shown in Figure 2. For the manganese-based samples, the following signals are observed: (i) stretching vibration of the Mn-O bonds in the MnO_6_ octahedrons appears between 500 and 786 cm^−1^, and (ii) asymmetric stretching of carbonate ions at 858 and 1443 cm^−1^ indicates the presence of BaCO_3_ [46,47,48]. For iron-based samples, in addition to carbonate ions that, according to the intensity of signals, are present in a higher amount than in manganese-based samples, the following signals are located: (i) peaks at wavenumbers slightly higher than 598 and 665 cm^−1^ that correspond to the stretching vibration of the Fe-O bonds of FeO_6_ octahedrons, indicating that the Fe-O bond is stronger than the Mn-O bond [49]; (ii) a signal placed at 768 cm^−1^ corresponding to a BaFe_2_O_4_ spinel, which is only detected for the B0.7F-E catalyst [49,50]; and (iii) a signal placed at 1060 cm^−1^ that corresponds to the stretching vibration of the C-OH bond [51,52,53]. The presence of carbon could be due to the remaining EDTA or citric acid, which was not completely removed during the calcination step. Note that as BaCO_3_ was not detected by XRD, it should be an amorphous phase.

### 2.2. Surface Properties

**Figure 3:**
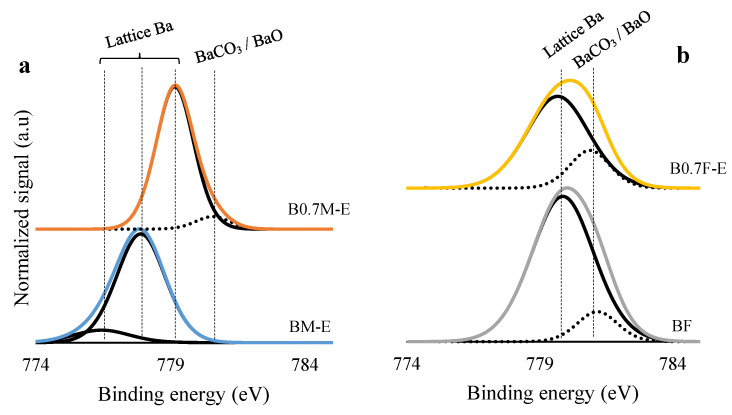
XPS spectra of the Ba 3d^5/2^ transition for BM-E and B0.7M-E (**a**) and for BF and B0.7F-E (**b**) catalysts.

XPS spectra were obtained to determine the surface chemical composition of the selected samples. The spectra of the Ba 3d^5/2^ transition for both catalysts series shown in Figure 3 reveal that lattice Ba is the main species on the surface and that the amount of BaCO_3_ (which was previously observed by FT-IR) is more abundant on the surface of iron-based catalysts than on manganese-based samples.

**Figure 4:**
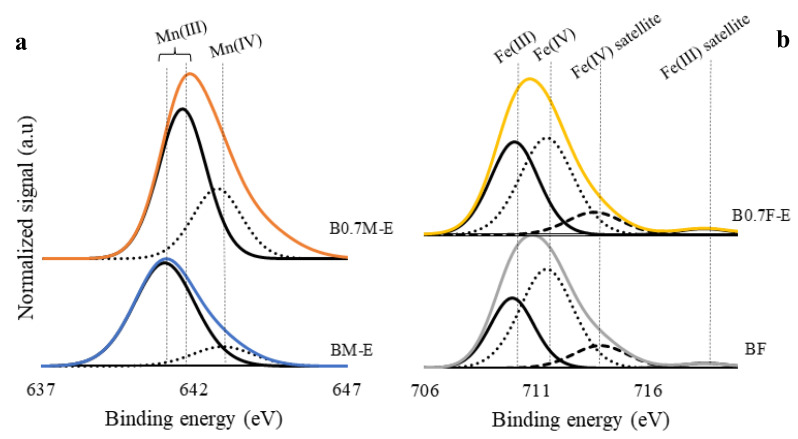
XPS spectra of the Mn 2p^3/2^ (**a**) and Fe 2p^3/2^ (**b**) transitions for BM-E, B0.7M-E, BF, and B0.7F-E catalysts.

XPS spectra corresponding to the Mn 2p^3/2^ transition are shown in Figure 4a. The registered signals are deconvoluted into two contributions associated with Mn(III) and Mn(IV), with Mn(III) located at lower binding energies than Mn(IV) [54]. The Mn(III)/Mn(IV) ratio values (presented in Table 3 and calculated by employing the corresponding area under the deconvoluted signals) reveal a higher amount of Mn(III) than of Mn(IV) on the catalyst surface and that the proportion of Mn(IV) increases as Ba content decreases. Thus, it seems that the oxidation of Mn(III) to Mn(IV) is the charge compensation mechanism working in this sample in order to counteract the decrease in the positive charge in barium-deficient perovskites [55].

In the Fe 2p^3/2^ transition spectra shown in Figure 4b, the following contributions can be identified: (i) an Fe(III) peak appearing at binding energies lower than those of Fe(IV) and (ii) Fe(IV) and Fe(III) satellite peaks located at approximately 713 eV and 718 eV, respectively [54,56,57]. The Fe(III)/Fe(IV) ratio values presented in Table 3 indicate that both Fe oxidation states coexist on the surface of the BF and B0.7F-E catalysts, with Fe(IV) as the main oxidation state for the two catalysts. However, as the Fe(III)/Fe(IV) ratio increases for B0.7F-E, the Fe(III) oxidation to Fe(IV) seems not to take place to compensate the positive charge deficiency as occurs for the B0.7M-E sample.

**Figure 5:**
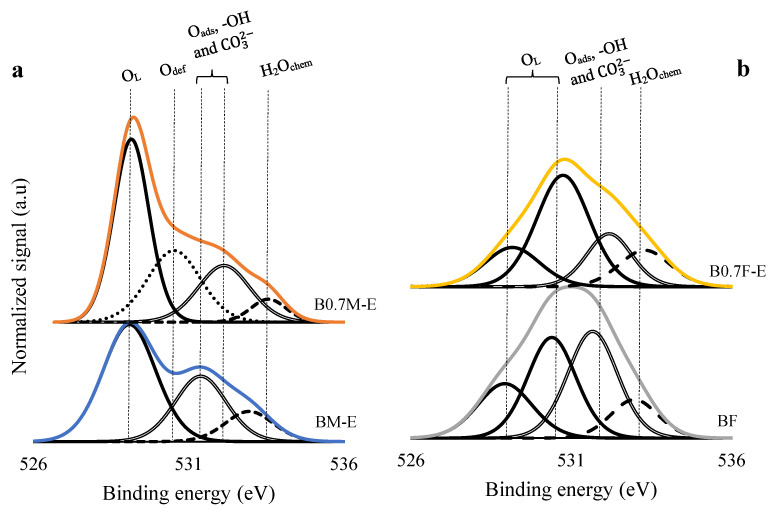
XPS spectra of the O 1 s transition for BM-E and B0.7M-E (**a**) and for BF and B0.7F-E catalysts (**b**).

Finally, the deconvolution of the O 1 s signal (Figure 5) shows three contributions: (i) a band located at binding energies around 529 eV, which is associated with the presence of lattice oxygen (O_L_) [54,58]; (ii) a band located around 531 eV that is related to the existence of defects with low oxygen coordination, that is, the oxygen vacancies formed on the surface (O_def_) [59]; (iii) a band detected at 532 eV that indicates the presence of adsorbed oxygen (O_ads_), hydroxyl (-OH), and carbonate (CO_3_^2−^) groups on the catalyst surface [54,58,60]; and, finally, (iv) a band at approximately 533 eV corresponding to chemisorbed water (H_2_O_chem_) [61,62]. For iron-based samples (Figure 4b), lattice oxygen peak shows two contributions corresponding to the lattice oxygen of the two main crystalline phases detected by XRD (BaFeO_2.67_ and BaFe_2_O_4_) at binding energies close to 531 eV and the oxygen of the BaFe_0.72_Fe_0.28_O_2.64_ minority phase (observed in XRD) at binding energies of approximately 529 eV. Table 3 shows the experimental values of the O_L_/(Ba + Mn/Fe) ratio (estimated using the areas under the corresponding deconvolutions) and the nominal values (calculated by employing the catalysts formulations of ABO_3_, A_0.7_BO_3_, or AB_2_O_4_). Thus, as the XPS ratios of BM-E, B0.7M-E, and BF are lower than the nominal ratios, the presence of oxygen vacancies on the surface of catalysts is featured. For manganese-based samples, the amount of oxygen vacancies increases as Ba content decreases, confirming the presence of a band corresponding to the oxygen defects (O_def_, located at around 531 eV) in B0.7M-E. Thus, it seems that the formation of these oxygen defects also enables counteracting the positive charge defect caused by the decrease in Ba content [63]. On the contrary, the formation of oxygen defects in the B0.7F-E sample is not promoted. Consequently, the change of structure from perovskite (in BF) to spinel (in B0.7F-E) seems to be the unique effect of Ba deficiency in iron-based catalysts.

### 2.3. Redox Properties

**Figure 6:**
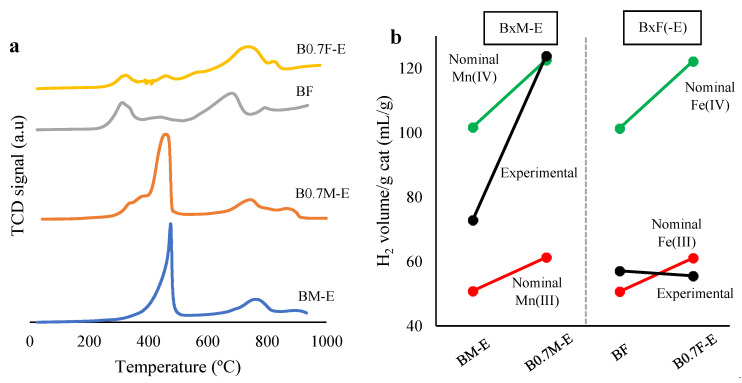
H_2_-TPR consumption profiles for catalysts (**a**) and H_2_ consumption (mL/g of catalyst) (**b**).

Reducibility and redox properties of the catalysts were analyzed using temperature-programmed reduction with H_2_ (H_2_-TPR), which represents the consumption profiles shown in Figure 6a. 

For barium manganese-based samples, three different peaks are identified [36,37,38,64,65,66,67,68]: (i) a low-temperature peak, which appears between 400 °C and 500 °C, assigned to the reduction of Mn(IV) and Mn(III) to Mn(II); (ii) a low-intensity signal at temperatures between 700 °C and 800 °C, which corresponds to the reduction of oxygen species on the catalysts surface; and (iii) another low-intensity band at around 900 °C due to the reduction of bulk Mn(III) to Mn(II). In the B0.7M-E sample, a small amount of hydrogen consumption is observed below 400 °C, which confirms the presence of Mn(IV) in the catalyst [69]. It is observed that the reduction of Mn(IV) and Mn(III) takes place at lower temperatures as the barium content decreases, probably due to a higher ionic mobility that improves the reduction process. This improved mobility has to be related to the presence of barium defects and oxygen vacancies in the structure [70]. 

On the other hand, for iron-based catalysts, the following peaks can be identified: (i) a peak at approximately 320 °C that corresponds to the reduction of Fe(IV) to Fe(III), (ii) a peak between 420 °C and 460 °C due to the partial reduction of Fe(III) to Fe(II), (iii) a third peak assigned to the reduction of the remaining Fe(III) between 650 °C and 740 °C, and finally, (iv) a peak at approximately 815 °C associated with the reduction of Fe(II) to Fe(0) [71]. It is observed that redox processes involving Fe cations take place at slightly lower temperatures in the BaFeO_2.67_ perovskite-type structure than in the BaFe_2_O_4_ spinel, which seems to be related to the higher amount of vacancies present in the oxygen-deficient perovskite than in the spinel structure, allowing a greater mobility and increased redox properties for the former.

In Figure 6b, the nominal and experimental consumptions of H_2_ are compared for both series of catalysts. Nominal consumptions were calculated considering that manganese (either as Mn(III) or Mn(IV)) and iron (either as Fe(III) or Fe(IV)) are reduced to Mn(II) and Fe(0), respectively. Experimental values were calculated from the area under the H_2_-TPR peaks identified in the H_2_-TPR consumption profiles. For manganese-based catalysts, as the experimental consumption is closer to the nominal value corresponding to Mn(IV), it seems that Mn(III) and Mn(IV) species coexist in the bulk of catalysts, with Mn(IV) as the main oxidation state for the B0.7M-E catalyst. This is in agreement with the presence of a higher amount of Mn(IV) on the surface of this catalyst, as revealed by the Mn(III)/Mn(IV) XPS ratio (Table 3). Note that the higher H_2_ consumption determined for the B0.7M-E catalyst with respect to the BM-E catalyst has to be also related to the higher amount of Mn present in this sample (see the chemical bulk composition in Table 2). However, as the normalized value of H_2_ consumption (i.e., the percentage of Mn shown in Table 2) is still higher for B0.7M-E (4.2) than for BM-E (2.9), it seems to be confirmed that this sample presents a higher reducibility due to the presence of a higher amount of more reducible Mn(IV). On the other hand, for the iron-based samples, as the experimental values are closer to nominal Fe(III) values, the amount of Fe(III) in the catalysts bulk is higher than that of Fe(IV), as was also deduced by XPS (see the Fe(III)/Fe(IV) ratio in Table 3) for surface composition.

### 2.4. O_2_ Release during Temperature-Programmed Desorption in He (O_2_-TPD)

**Figure 7:**
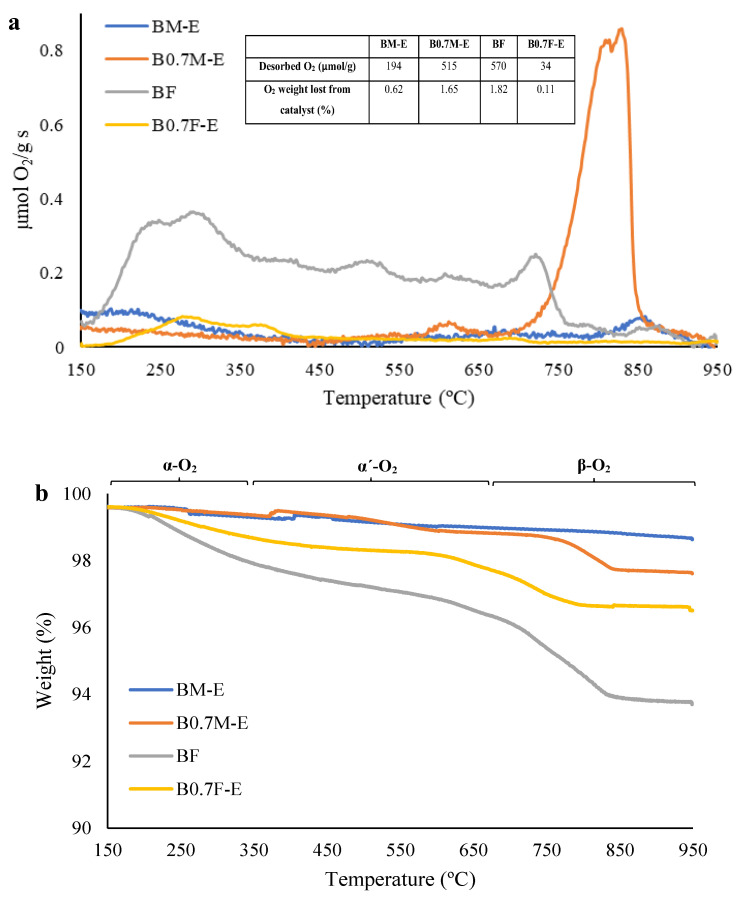
O_2_-TPD profiles (**a**) and weight profiles (**b**) of catalysts.

In the O_2_-TPD profiles featured in Figure 7a, three different peaks can be identified [72,73]: (i) between 150 °C and 350 °C, corresponding to the desorption of the oxygen adsorbed on surface vacancies (called α-O_2_); (ii) from 350 °C to 700 °C, the oxygen evolved comes from the adsorbed on the lattice defects (designated as α´-O_2_); and (iii) above 700 °C, where the release of lattice oxygen (β-O_2_) is detected. This β-oxygen is related to the reduction of Mn(IV)/Fe(IV) to Mn(III)/Fe(III) and the presence of oxygen vacancies in the bulk, which provides information about the oxygen mobility through perovskite lattice, which is a key factor determining the catalytic performance during oxidation reactions [74]. In Figure 7a, the total amount of desorbed oxygen and the weight loss percentage due to oxygen release for each catalyst are included. 

Note that for manganese-based perovskites, the desorbed O_2_ corresponds mainly to lattice oxygen (β-O_2_), although the BM-E catalyst also presents a low α-O_2_ emission and B0.7M-E shows a small O_2_ desorption peak at around 600 °C. Furthermore, the amount of labile oxygen released is notably higher for the barium-deficient perovskite (B0.7M-E) than for BM-E because B0.7M-E presents a higher amount of Mn(IV) than BM-E. The high mobility of oxygen allows a greater reducibility of the catalysts, as shown by H_2_-TPR results (see Figure 6a) [73]. On the other hand, iron-based samples mainly evolve oxygen below 750 °C in the form of α-O_2_ and α´-O_2_ species [75]. Note that in this case, the sample evolving the highest amount of oxygen is the BF catalyst, as expected for an under-stoichiometric perovskite, which is the main crystalline phase for this sample (see Figure 1a). Note that the structural change from perovskite to spinel that occurs due to the decrease in barium content in B0.7F-E does not increase the amount of oxygen vacancies nor the amount of Fe(IV). 

Figure 7b shows the weight loss profiles registered during O_2_-TPD experiments for the two series of catalysts. Different contributions are identified: (i) below 150 °C, the weight loss is due to the release of chemisorbed water; (ii) from 150 °C to 450 °C, the α-O_2_ species are mainly released; (iii) from 650 °C to 850 °C, α´-O_2_ and β-O_2_ are emitted; and finally, (iv) at approximately 815 °C, BaCO_3_ (previously detected by FT-IR and XPS) decomposition takes place [76]. The weight loss of manganese-based samples due to O_2_ release (Figure 7a) matches with the corresponding TGA profiles shown in Figure 7b. However, for iron-based catalysts, a higher total weight loss than that expected according to the evolved O_2_ (data shown in Figure 7a) is detected due to the decomposition of carbonate groups present in a higher amount in iron-based samples than in manganese-based samples (see XPS and IR results).

### 2.5. Catalytic Activity

The effect of decreasing the Ba content on the catalytic performance of BM and BF perovskites for CO and soot oxidation in simulated GDI engine exhaust conditions was analyzed.

**Figure 8:**
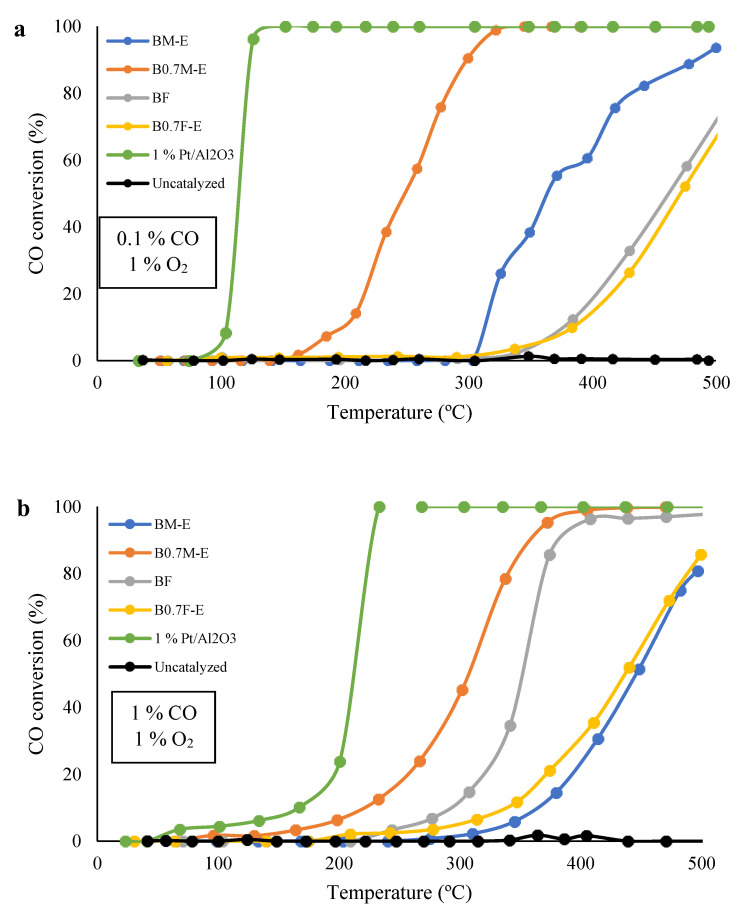
CO conversion profiles (CO-TPR) for the catalysts in 0.1% CO, 1% O_2_ in He (**a**) and 1% CO, 1% O_2_ in He (**b**).

Figure 8 features the CO conversion profiles for the two tested reaction atmospheres, and Table 4 presents the temperature at which 50% of CO conversion was reached (T_50%_ values). The CO conversion profiles reveal that all catalysts are active for CO oxidation under the tested conditions, as CO is not oxidized in the absence of a catalyst. However, all catalysts are less active than the 1% Pt/Al_2_O_3_ used as reference catalyst.

For the low CO content gaseous mixture (0.1% CO, 1% O_2_), Figure 8a indicates that manganese-based catalysts are more active than the iron-based catalysts, as Mn is more efficient than Fe in terms of promoting redox processes due to a greater redox versatility [77]. Note that for manganese-based samples, Ba deficiency improves CO conversion at low temperatures, since the B0.7M-E catalyst presents a higher amount of oxygen vacancies (that are active sites for the reaction), a higher amount of Mn(IV), and a higher reducibility than BM-E perovskite. On the contrary, almost no effect of Ba deficiency is detected for iron-based samples because a structural change from perovskite to spinel occurs in this sample instead of an increase in the amount of oxygen vacancies and/or in the amount of Fe(IV). In fact, B0.7F-E presents a higher T_50%_ value than BF (see Table 4), suggesting that a spinel structure, which is the main crystalline phase for B0.7F-E, is less active than a perovskite structure for the CO oxidation reaction because of its lower amount of oxygen vacancies and Fe(IV) with respect to BF with a perovskite structure (see XPS and H_2_-TPR data in Table 3 and Figure 5b and Figure 6) [78].

On the other hand, for a stoichiometric gaseous reactant mixture (1% CO, 1% O_2_), Figure 8b shows that the B0.7M-E sample is more active than the BF sample. Therefore, manganese-based mixed oxides with perovskite structures are more active than iron-based mixed oxides with perovskite structures (BF) for CO oxidation under the two tested conditions. This is because manganese-based perovskite presents a higher amount of active sites for CO oxidation than iron-based perovskite, as it presents not only a high fraction of Mn(IV) but also a higher amount of oxygen vacancies. Therefore, it is confirmed that the nature of the B cation in perovskite determines the catalytic performance in oxidation reactions. In this sense, it is well known that electronic configuration of the B cation in perovskite is a key factor for adsorption/activation of reactants (CO and O_2_ in this case). In fact, the occupancy of the e_g_ orbital (which presents the ideal geometry for interacting with the molecular orbitals of CO and O_2_ molecules) establishes the adsorption/desorption energy of reactants [79], as the energy of adsorption increases as the number of electrons in the e_g_ orbital decreases. Therefore, as Fe(III) has two e_g_ electrons but Mn(III) has only one (and Mn(IV) presents an empty e_g_ orbital), the CO adsorption energy is higher for manganese perovskites than for iron perovskites. Consequently, manganese allows a more efficient CO activation on the catalyst surface, promoting Langmuir–Hinshelwood (LH) and Eley–Rideal (ER) mechanisms. Additionally, as the amount of oxygen vacancies is higher for manganese perovskites than for iron perovskite, a higher number of active sites is present in the former samples. Note that the prevalence of LH or ER mechanisms mainly depends on the gas mixture composition [59].

Additionally, the data presented in Table 4 shows that the increase in the CO partial pressure has a different effect for manganese than for iron-based samples, i.e., the CO conversion decreases for the former but improves for the latter. The observed decrease in the catalytic activity for manganese-based samples indicates that the higher demand of active sites at higher reactant partial pressure only seems to be achieved at higher temperatures. On the other hand, the increase in the CO conversion at higher CO partial pressures observed for the BF catalyst reveals the participation of α-O_2_ and α´-O_2_ present (detected during O_2_-TPD experiments, see Figure 7a) as active species. In fact, the amount of oxygen evolved by BF and B0.7F-E samples at temperatures below 700 °C (included in Figure 7a) is in agreement with the ΔT_50%_ values presented in Table 4. Finally, by comparing ΔT_50%_ for B0.7M-E and B0.7F-E catalysts with that observed for the 1% Pt/Al_2_O_3_ reference sample, it seems that mixed oxide catalysts are more stable than Pt-based catalysts against fluctuations in the composition of the reactant mixture, so they are more resistant to changes in the amount of O_2_ and CO in the exhaust evolved by GDI engines, which seems to be an advantage for a potential application.

**Figure 9:**
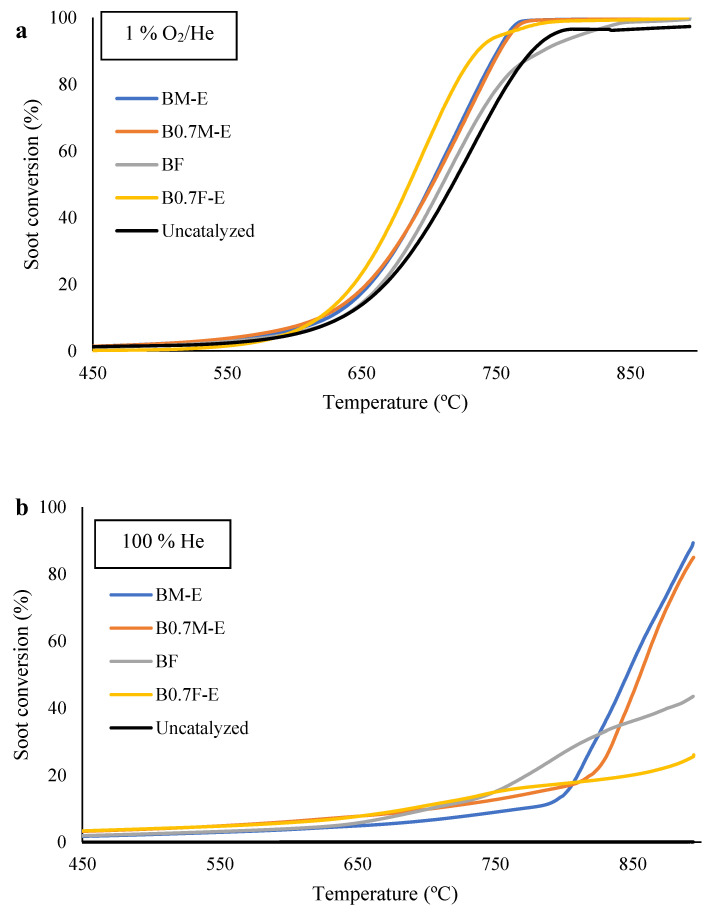
Soot conversion profiles (soot-TPR) for the catalysts in 1% O_2_ in He (**a**) and in 100% He (**b**).

The soot conversion profiles obtained during the soot oxidation experiments carried out under the simulated conditions close to those of GDI engine exhaust, i.e., an inert atmosphere and a 1% O_2_/He gas mixture, are featured in Figure 9a,b. T_25%_, T_50%_ (temperatures required to achieve the 25% and the 50% of soot conversion, respectively) and the selectivity to CO_2_ are featured in Table 5. The data confirm that the two types of catalysts are also able to increase the soot conversion in the two tested atmospheres, as soot oxidation takes place at temperatures lower than for an uncatalyzed reaction. However, the improvement in the soot conversion is lower than that observed for the CO oxidation reaction, being the increase in the selectivity to CO_2_ more relevant than the decrease in T_25%_ and T_50%_ values. 

In 1% O_2_/He, the B0.7F-E sample presents the lowest T_50%_ value, so it seems that the spinel structure of B0.7F-E catalysts presents a higher ability to adsorb and dissociate O_2_ from the gas phase than a perovskite structure [80]. 

In the absence of oxygen, as expected [43,81,82], all catalysts show lower soot conversions due to the lower amount of oxygen available (which only comes from the samples). Under these conditions, the BF sample shows the lowest T_25%_, as it evolves the highest amount of low and intermediate oxygen species (α-O_2_ and α´-O_2_; see Figure 7a) able to oxidize the soot. However, at temperatures higher than around 800 °C, the conversion in the presence of manganese notably increases, and, consequently, lower T_50%_ values are shown for manganese-based samples than for iron-based samples. This behavior seems to be related to the higher emission of β-O_2_ able to oxidize soot.

On the other hand, the selectivity to CO_2_ values shown in Table 5 reveal that the generation of CO_2_ is highly dependent on the catalyst and on the atmospheric composition. As expected [43,80], the selectivity values in the presence of oxygen in the reaction atmosphere are higher than in inert atmosphere, as in the latter, less oxygen is available for the oxidation of soot. Note that in the presence of O_2,_ the increase in the selectivity to CO_2_ is higher for manganese than for iron-based samples. This is in agreement with the higher ability of Mn with respect to Fe to catalyze the CO oxidation to CO_2_, as previously discussed. Finally, it is remarkable that in an inert atmosphere, the Fe based catalysts present a higher selectivity to CO_2_ than Mn-based catalysts because the latter metal promotes soot oxidation at higher temperatures, at which CO is the main product of carbon (soot) oxidation [83]. 

Thus, it can be concluded that the role of Ba_x_BO_3_ samples as catalysts in oxidation reactions depends on the reaction (CO or soot oxidation) and on the nature of the B cation, as Mn-based are samples more active for CO oxidation, with B0.7F-E showing a higher increase in soot conversion in the presence of oxygen, even though the catalytic effect is more significant for the CO oxidation reaction. Additionally, it has been observed that the decrease in the Ba content improves the catalytic performance of both catalysts, as B0.7M-E is more active than BM-E for the catalysis of CO oxidation, as B0.7F-E is a better catalyst than BF for soot conversion under simulated GDI engine exhaust conditions. This shows that the composition of BaBO_3_ mixed oxides can be tailored as a function of the oxidation reaction that has to be catalyzed.

Finally, considering that the four samples studied present a moderate ability as catalysts for CO and soot oxidation compared with other noble-metal-free catalysts [83,84,85], further optimization of perovskite composition is mandatory. In this sense, it is well known that copper plays an important role as an active phase during CO and soot oxidation reactions [84,85,86]. Consequently, the next step will be the analysis of the effect of the decrease in the barium content in copper–manganese perovskites [73].

## 3. Materials and Methods

### 3.1. Synthesis and Characterization of Catalysts

The Ba_x_MnO_3_ and Ba_x_FeO_3_ perovskites were synthesized by employing the sol–gel method adapted to aqueous medium [80,87]. First of all, a 40 mL solution (containing EDTA as a chelating agent in a 1:2 molar Mn/Fe:EDTA ratio) was prepared at 60 °C. Ba(NO_3_)_2_ was added as a Ba precursor; then, Mn(NO_3_)_2_·4 H_2_O or Fe(NO_3_)_3_·9 H_2_O was included as an Mn or Fe precursor, respectively. After that, citric acid was incorporated using a 1:2, Mn(Fe):citric acid molar ratio. Subsequently, the temperature was increased to 80 °C to assure gel formation. The described procedure was carried out keeping the pH at 9 using an ammonia solution (30% wt). Finally, the obtained gel was dried at 150 °C for 12 h, and as the last step, the solid was calcinated at 850 °C for 6 h. Additionally, in an attempt to decrease the number of crystalline phases present in the samples, Ba_x_FeO_3_ catalysts were synthesized using the same method but avoiding the use of EDTA (named BF and B0.7F). Thus, even though the synthesis method was the same, some experimental variables were slightly modified with respect to the procedure described above: (i) citric acid was dissolved in a 1:2 molar Mn (Fe):citric acid ratio before adding the metal precursors, (ii) barium acetate was used instead of barium nitrate, (iii) the gelation process was conducted at 65 °C, and (iv) the drying process was carried out at 90 °C for 48 h. 

For sample characterization, the following techniques were employed.

The chemical bulk composition was analyzed by μ-XRF on an Orbis EDAX analyzer. 

The textural properties were determined by N_2_ adsorption at −196 °C using an Autosorb-6B instrument from Quantachrome (Anton Paar Austria GmbH). The samples were degassed at 250 °C for 4 h before the N_2_ adsorption experiments. 

The crystalline structure was obtained using X-ray diffraction (XRD). The X-ray patterns were recorded between 20º and 80° 2θ angles with a step rate of 0.4°/min and using Cu K_α_ (0.15418 nm) radiation in a Bruker D8-Advance device. FT-IR spectra from 4000 to 500 cm^−1^ were also obtained using a BRUKER IFS 66/S apparatus with a spectral resolution of 4 cm^−1^.

Surface chemistry was studied by X-ray photoelectron spectroscopy (XPS) using a K-Alpha photoelectron spectrometer by Thermo-Scientific with an Al K_α_ (1486.7 eV) radiation source. To obtain XPS spectra, the pressure of the analysis chamber was maintained at 5 × 10^−10^ mbar. The binding energy (BE) and kinetic energy (KE) scales were adjusted by setting the C 1 s transition to 284.6 eV, and the BE and KE values were determined with the peak-fit software of the spectrometer. The O_L_/(Ba + Mn/Fe), Mn(IV)/Mn(III), and Fe(IV)/Fe(III) XPS ratios were calculated using the area under the suggested deconvoluted bands of O 1 s, Mn 2p^3/2^, Fe 2p^3/2^, and Ba 3d^5/2^.

The reducibility of catalysts was determined by temperature-programmed reduction with H_2_ (H_2_-TPR) in a Pulse Chemisorb 2705 (from Micromeritics) provided by a thermal conductivity detector (TCD) and using 30 mg of sample heated at 10 °C/min from 25 °C to 1000 °C in a 5% H_2_/Ar atmosphere (40 mL/min). Quantification of the H_2_ consumption was carried out using a CuO reference sample.

O_2_-TPD experiments were performed in a TG-MS (Q-600-TA and Thermostar from Balzers Instruments (Pfeiffer Vacuum GmbH, Germany), respectively) with 16 mg of sample heated at 10 °C/min from room temperature to 950 °C under a 100 mL/min helium atmosphere. Moreover, all the samples were preheated to 150 °C for 1 h for moisture removal. The 18, 28, 32, and 44 m/z signals were registered for the H_2_O, CO, O_2_, and CO_2_ evolved during the experiments, respectively. The amount of evolved oxygen was estimated using a CuO reference sample, and the weight data recorded by the TG system were used to obtain the mass loss profiles.

### 3.2. Activity Tests

To determine the catalytic activity for CO oxidation, temperature-programmed reaction (CO-TPR) experiments were developed using two reactant mixtures composed of (i) 0.1% CO and 1% O_2_ in He (low CO reactant mixture) and (ii) 1% CO and 1% O_2_ in He (near-stoichiometric reactant mixture, which simulates the actual working conditions of GDI engines). For the experiments, 50 mg of catalysts and 100 mg of SiC were loaded into a U-shaped quartz reactor, and a 10 °C/min heating rate was used from room temperature to 500 °C. The mixture catalyst-SiC was preheated for 1 h at 600 °C in a 5% O_2_/He gas mixture in order to clean the surface of the catalysts. For quantification of reaction products, a HP6890 gas chromatograph equipped with a thermal conductivity detector and two packed columns (Porapack-Q and MolSieve-13X) was employed.

The CO conversion was determined using the following equation.

CO conversion (%) = ((CO_in_ − CO_out_)/CO_in_) · 100 (1)

The catalytic tests for the soot oxidation reaction under simulated GDI conditions were performed in the TG-MS system used for O_2_-TPD experiments. To develop these tests, 16 mg of a catalyst and soot mixture (soot:catalyst ratio of 1:8, using Printex-U as model soot in loose contact mode) was preheated for 1 h at 150 °C in a 1% O_2_/He gas mixture (100 mL/min); then, the temperature was increased at 10 °C/min until 900 °C (soot-TPR). Two different reactant atmospheres were employed: (i) 1% O_2_/He, which simulates “fuel cuts” GDI exhaust conditions, and (ii) 100% He, which reproduces regular stoichiometric GDI operations [43]. 

Soot conversion and the selectivity to CO_2_ were estimated using the following equations:

Soot conversion (%) = ((Σ_0_^t^(CO_2_ + CO))/Σ_0_^final^(CO_2_ + CO)) · 100 (2)

Selectivity to CO_2_ (%) = (CO_2,total_/Σ_0_^final^(CO_2_ + CO)) · 100 (3)

where Σ_0_^t^(CO_2_ + CO) is the amount of CO_2_ and CO evolved at time t, while Σ_0_^final^(CO_2_ + CO) is the total amount of CO + CO_2_ evolved during the test coming from the oxidation of the total amount of soot.

## 4. Conclusions

In this study, two series of Ba_x_MnO_3_ and Ba_x_FeO_3_ (x = 1 and 0.7) catalysts were synthetized, characterized, and tested for CO and soot oxidation reactions under simulated GDI exhaust conditions. Based on the results discussed above, the following conclusions can be exposed:

(1) Manganese-based samples present a BaMnO_3_ hexagonal perovskite structure, as the decrease in the amount of Ba does not significantly modify the crystalline structure. For iron-based samples, the decrease in the Ba content promotes the transition from a BaFeO_2.67_ hexagonal perovskite structure to a BaFe_2_O_4_ spinel structure.
(2) To compensate for the Ba deficiency in the manganese-based samples, the amount of Mn(IV) and the oxygen vacancies increases, allowing a higher reducibility and oxygen mobility. In iron-based samples, the Ba deficiency only causes a change in the structure from perovskite to spinel.
(3) Manganese-based perovskites (BM-E and B0.7M-E) show a better catalytic performance than iron-based perovskite (BF) for CO oxidation reactions due to the higher generation of actives sites.
(4) The decrease in the Ba content improves the catalytic performance of both catalysts, as B0.7M-E is more active than BM-E for CO oxidation, and B0.7F-E presents a higher activity for soot conversion than BF under simulated GDI engine exhaust conditions. This shows that the composition of BaBO_3_ mixed oxides can be tailored as function of the oxidation reaction to be catalyzed.

## Figures and Tables

**Table 1:** XRD data for BM-E, B0.7M-E, BF, and B0.7F-E catalysts.

Catalyst	Average Crystal Size (nm) ^1^	$\mathrm{Cell}\;\mathrm{Parameters}\;(\dot{A})$		
		a	b	c
BM-E	24.76	5.69	5.69	4.81
B0.7M-E	18.24	5.69	5.69	4.81
BF	49.52	5.67	5.67	13.96
B0.7F-E	23.09	19.02	5.38	8.48

^1^ Average crystal sizes for the hexagonal perovskite phase, except for B0.7F-E, which corresponds to a spinel phase.

**Table 2:** Chemical bulk composition and specific surface area data for BM-E, B0.7M-E, BF, and B0.7F-E catalysts.

Catalyst	BET Surface Area (m^2^/g)	Chemical Composition (wt %)					
		Experimental			Nominal		
		Ba	Mn or Fe	O	Ba	Mn or Fe	O
BM-E	9	62	24	14	57	23	20
B0.7M-E	11	55	30	15	48	28	24
BF	9	59	24	17	58	24	18
B0.7F-E	10	51	30	19	44	36	20

**Table 3:** XPS data for BM-E, B0.7M-E, BF, and B0.7F-E catalysts.

Catalyst	Mn(III)/Mn(IV)	Fe(III)/Fe(IV)	XPS O_L_/(Ba + Mn/Fe) (Nominal)
BM-E	5.3	-	1.2 (1.5)
B0.7M-E	2.1	-	1.2 (1.8)
BF	-	0.6	1.1 (1.5)
B0.7F-E	-	0.9	1.4 (1.3 ^1^)

^1^ Calculated based on the spinel formula (BaFe_2_O_4_).

**Table 4:** T_50%_ values for the tested catalysts and T_50%_ change (ΔT_50%_) in low CO content mixture (0.1% CO, 1% O_2_) with respect to the stoichiometric mixture (1% CO, 1% O_2_).

Catalyst	T_50%_ (0.1% CO, 1% O_2_) (°C)	T_50%_ (1% CO, 1% O_2_) (°C)	ΔT_50%_ (°C)
BM-E	364	446	82
B0.7M-E	249	307	58
BF	461	352	−109
B0.7F-E	471	437	−34
1% Pt/Al_2_O_3_	114	212	98

**Table 5:** T_25%_, T_50%_, and selectivity to CO_2_ (S_CO_2__) values for BM-E, B0.7M-E, BF, and B0.7F-E catalysts.

Catalyst	1% O_2_/He			100% He		
	T_25%_ (°C)	T_50%_ (°C)	S_CO_2__ (%)	T_25%_ (°C)	T_50%_ (°C)	S_CO_2__ (%)
BM-E	666	701	67	816	847	8
B0.7M-E	665	702	78	830	855	11
BF	674	710	71	794	-	42
B0.7F-E	653	686	63	892	-	33
Uncatalyzed	679	718	42	-	-	-

## Data Availability

Not applicable.
